# Supplementary material for: Pre‐clinical validation of a selective anti‐cancer stem cell therapy for Numb‐deficient human breast cancers
Source: EMBO Mol Med. 2017 Mar 15;9(5):655–71. doi: 10.15252/emmm.201606940 (PMC5412856; doi:10.15252/emmm.201606940)
Supplement: Supplementary file 1 — Appendix [file EMMM-9-655-s001.pdf]

APPENDIX

TABLE OF CONTENTS

- Legends to Appendix Figures S1-S5

- Appendix Supplemental Methods

- Appendix Figure S1

- Appendix Figure S2

- Appendix Figure S3

- Appendix Figure S4

- Appendix Figure S5

APPENDIX FIGURE LEGENDS

Appendix Figure S1. Additional characterization of tumors and PDXs used in the study.

A. MSs derived from the indicated Numb$^-$ and Numb$^+$ tumors were treated *in vitro* with the proteasome inhibitor MG132 (0.5 μM for 48 h) and analyzed by IB as indicated. The increase in β-catenin was used as a control for the efficacy of proteasome inhibition by MG132. Panels shown in the IB were assembled either using lanes from the same blot (splicing out lanes loaded with additional controls or non-relevant samples), or from different blots run and stained simultaneously (see also Materials and Methods). GRP94, loading control.

B. Top. Representative images of Numb IHC staining (brown) of haematoxylin-eosin counterstained FFPE sections of Numb$^-$ (T3 and T4) and Numb$^+$ human BCs (TC and TD). Scale bar = 200 μm. Bottom, the four tumors were orthotopically xenografted into NGS immunocompromised mice and resulting PDXs were analyzed for Numb expression by IHC staining. Scale bar = 200 μm.

C. Top, representative images of T1 and N1 MS. Scale bar = 100 μm. Middle, average MS size (± SD of triplicates) calculated as the ratio of total number of cells to MS numbers at passage 2 and 3 of the serial propagation assay. Bottom, MS average diameter (± SD), calculated at passage 2 of the serial propagation assay, on 10 N1 and 10 T1 MSs. Similar results were obtained for N2 and T2, which represent matched normal and tumor counterpart from a different patient (not shown). Additional 20 normal samples were also analyzed, showing behaviour similar to N1 and N2 (not shown).

D. Cells dissociated from first generation MSs obtained from a normal (N1, left) or tumor (T1, middle) sample from the same patient were plated in triplicate at a density of 5,000 cells/ml in 24-well plates to yield second MS generation. MSs were counted after 7 days. The same procedure was repeated for 6 serial passages. The cumulative sphere number over six passages (± SD of triplicates) is reported. The experiment is representative of three biological replicas. In the rightmost panel, the same data are shown on a semilogarithmic scale. Trend lines (light lines) that best approximate the curves were obtained by regression analysis. R^2, coefficient of determination; GR, growth rate (derived from the equation of the exponential curve $y=Ne^{GX}$ that passes through the different points). The analysis of the replicative potential of N1-MFCs reveals that the total number of N1-MSs decreased progressively at each passage, until exhaustion after four to six passages. The cumulative MS number approximated an exponential curve (R2 = 0.99) with a growth rate (GR) of 0.51, indicating a decrease rate of approximately 50% at each passage. Of note, the average size of N1-MSs remained constant throughout the passages (see panel C, middle), thus suggesting that N1 SCs, maintain the same growth potential. The number of T1-MSs, instead, increased at every passage, with a constant 5-fold expansion (GR= 5.8), suggesting that T1-MFCs are nearly immortal (up to 30 passages in selected experiments). Together, these findings recapitulate the results previously obtained in the mouse mammary gland (Cicalese *et al.*, 2009, Tosoni *et al.*, 2015) according to which

normal SCs rapidly loose self-renewal potential *in vitro*, whereas Numb-negative CSCs are nearly immortal.

E. A typical serial propagation experiment with MSs derived from the T3, T4, TC and TD tumors. The cumulative sphere number following serial replating of MSs over four passages is shown.

F. Example images of MSs from a Numb$^-$ (T1) and a Numb$^+$ (TA) BC at passages 2 and 4 in the MS propagation assay. Scale bar = 150 μm.

Appendix Figure S2. Effects of Numb re-expression on mode of division of Numb$^-$ human breast CSCs.

A. Methodology used for establishing the mode of division (symmetric *vs.* asymmetric) of breast CSCs. We used the retrospective method for the assessment of mode of division, as extensively described in (Cicalese *et al*, 2009; Tosoni *et al*, 2015). Briefly, MECs obtained from human tumors were cultured in non-adherent conditions, as described (Cicalese *et al*, 2009; Pece *et al*, 2010; Tosoni *et al*, 2012). Under these conditions, as shown on the left, most MECs do not proliferate or they undergo *anoikis* (represented by dashed circles), while SCs give rise to MSs (represented by clusters of solid circles). The formation of MSs was monitored for 7-10 days by time-lapse video microscopy. The modality of the first SC division (shown on the right) was established by following the pattern of cell number progression within the forming MS. A 1-2-3-5 pattern of cell number progression (where the 3-cell stage lasts at least 2 hours) was considered as the result of an initial asymmetric division of the SC, followed by symmetric divisions of the progenitors, while the SC withdrew into quiescence. Conversely, a 1-2-4-6/8 progression pattern was the result of an initial symmetric division of the SC. Importantly, most cells from the primary MEC cultures (the progenitors) did not give rise to secondary MSs, or generated only small spheroids (up to 30 cells), which were not considered

in the analyses shown in the main text. Thus, only spheroids, equal or larger than 70 μM in size, were considered as MSs and analyzed retrospectively in the time-lapse movies.

B. Representative time-lapse video microscopy of a MS generated by MECs from the T1 Numb^{-} tumor. Panels: T1 Ctr, formation of a MS (and symmetric mode of division) by T1 cells; T1+Nb, formation of a MS (and asymmetric mode of division) by T1 cells transfected with Numb-dsRed; Epifluorescence, epifluorescence of T1+Nb cells as above. Note how Numb-dsRed segregates at one pole of the cell just before the first mitotic division, and note also the asymmetric partitioning of Numb at the first mitotic division into one of the daughter cells, which represents the hallmark behaviour of Numb during asymmetric self-renewing division of normal mammary SCs (Tosoni *et al*, 2015). Scale bar = 10 μm. Dashed lines indicate the perimeters of the cells.

C. Left. Numb-GFP-infected (green) and PKH-labelled (PKH26, red), normal MECs, were plated in non-adherent condition in methylcellulose and analyzed by time-lapse video-microscopy to monitor the first two mitotic divisions. Elapsed time is indicated. The black dashed lines indicate the first and second mitotic division, respectively, while the white dashed lines indicate the perimeters of the cells and of the newly formed MS. Arrowheads point to the stem-daughter cell, identified as the not dividing daughter cell (at the 2nd SC mitotic division). Note that this cell retained the Numb protein also at three-cell stage. PKH26 (Pece *et al*, 2010; Tosoni *et al*, 2015) was used to identify the low-proliferating daughter cell (i.e. SC). Scale bar = 10 μm.

D. Top, quantification of the mode of PKH-labelled SCs divisions; bottom, quantification of Numb partitioning at the first mitotic division mitosis. Data are from a minimum of 30 cells (from five independent experiments) performed as in (C).

Appendix Figure S3. Specificity controls for the effects of Nutlin-3.

We performed a series of specificity controls for potential toxic and or off-target effects of Nutlin-3, using the inactive enantiomer, Nutlin-3b.

A. MS from a Numb$^-$ tumor (T1) were treated with 10 μM Nutlin-3 (Nutl.), or with the indicated concentrations of the enantiomer Nutlin-3b (Enantiomer, En.), and subjected to IB (left) or Q-PCR (right). The samples Ctr, refer to treatment with vehicle (DMSO) only. As a control for toxicity, cells were also treated with Cisplatin (Cis., 40 μM). Nutlin-3, at the dose employed in our experiments (10 μM), did not affect the levels of the proliferation marker Ki67or of the apoptosis marker, activated caspase-3 (Activ. casp.-3). In contrast, treatment with cisplatin resulted in strong inhibition of Ki67 expression and elevated levels of activated caspase-3. In addition, the enantiomer Nutlin-3b did not show appreciable effects (compare the effects on the p53 target genes, mdm2 and p21, in the Q-PCR analysis on the right) at doses up to 5 times higher than the dose of Nutlin-3 employed. In the IB, vinculin was used as a loading control. Panels shown in the IB were assembled from different lanes of the same blot by splicing out lanes loaded with additional controls.

B. MS-forming ability of MECs from the indicated tumors, treated with the indicated doses of Nutlin-3 (Nutl.) or Nutlin-3b (En.) *vs.* vehicle-treated control cells (Ctr). Data are mean values of three independent experiments (± SD of 9 measurements), and are expressed relative to the SFE in Ctr cells (=100%). Unpaired two-sided Student's t-test. *, $P < 0.05$ *vs.* Ctr was considered as significant (T1, P = 9.76E-09; T2, P = 2.12E-07).

Also in this assay, Nutlin-3b was inactive at concentrations 5 times higher than the concentration of Nutlin-3 used in our experiments.

C. MS from the Numb$^-$ T1 tumor were treated *in vitro* with 10 μM Nutlin-3 (Nutl.), or the indicated concentrations of Nutlin-3b (En.) or vehicle (Ctr), and tested for tumorigenicity by orthotopic transplantation in immunocompromised NGS mice. Bars represent the tumor volume, expressed as the mean value of three independent experiments (± SD of a minimum of 16 to a maximum of 18 tumors). Unpaired two-sided Student's t-test. A P value < 0.05 was

considered as significant (*, *P vs.* Ctr = 6.80E-14). No detectable effects of Nutlin-3b on tumor growth were observed.

Appendix Figure S4. Characterization of Numb$^-$ and Numb$^+$ tumors used in *in vivo* efficacy studies to test the effects of Nutlin-3 and Paclitaxel in a combinatorial regimen

A. Representative images of Numb IHC staining (brown) performed on haematoxylin-eosin counterstained FFPE sections of Numb$^-$ (T5, T6 and T7) and Numb$^+$ (TE) human BCs. Scale bar = 200 μm.

B. Clinical and pathological features of Numb$^-$ (T5, T6 and T7) and Numb$^+$ (TE) human BCs used for *in vivo* efficacy studies described in Fig 7.

C. Quantification of the IHC analysis for Ki67 and activated caspase-3 expression performed in T5, T6, T7 Numb$^-$ and in TE Numb$^+$ tumors at the end of the dosing period (see Material and Methods and legend to Fig 7 for further details on the doses and treatment schedules employed for Nutlin-3 and Paclitaxel, alone or in combination). Data express the percentage of Ki67- or activated caspase-3-positive cells (n >10,000 cells counted for at least three tumors for each treatment condition).

D. Representative IHC images of Ki67 and activated caspase-3 (activated casp-3) of T5 Numb$^-$ and TE Numb$^+$ tumors. Scale bar = 100 μm.

Appendix Figure S5. Effects of the *in vivo* treatment with Nutlin-3 and Paclitaxel, alone or in combination, on the CSC content of Numb$^-$ and Numb$^+$ tumors.

Quantification of ALDH+ or CD44$^+$/CD24$^-$ cells was performed at the end of a 15-day treatment with Nutlin-3 or Paclitaxel alone, or with a combination of Nutlin-3+Paclitaxel (same experimental setting of the *in vivo* efficacy studies described in Fig 7). Randomly selected tumors explanted from the indicated Numb$^-$ and Numb$^+$ PDX-bearing mice were digested and the resulting cell populations were subjected to FACS analysis to measure the

distribution pattern of $CD44^+/CD24^-$ and ALDH+ cells, in the absence of any further treatment. In accordance to results from the functional *in vitro* and *in vivo* assays for CSC activity (see Fig 7), these cytofluorimetry studies support the conclusion that Nutlin-3 selectively affects, in $Numb^-$ but not $Numb^+$ tumors, the CSC compartment *in vivo*. There are however a number of caveats that may limit the usage of the two configurations that we used ($CD44^+/CD24^-$ and ALDH+ status) as universal breast CSC markers, raising the question as to which marker configuration is optimal for identification of CSCs in the clinical setting. We noted, for instance, that the $CD44^+/CD24^-$ and ALDH+ profiles identify different percentages of putative CSCs even in the same tumor, a finding in keeping with the notion that there is often little overlap of these markers in the identification of breast CSCs (Ginestier *et al*, 2007; Ricardo *et al*, 2011). Another important observation is that, in keeping with previous studies (Ricardo *et al*, 2011), the molecular characteristics of the tumor appear to impact on the marker profile of CSCs, as argued for by evidence that the luminal-type, estrogen receptor-positive $Numb^-$ T7 tumor displays almost undetectable levels of $CD44^+/CD24^-$ cells compared to the basal-type, estrogen receptor-negative $Numb^-$ T5 tumor (see Appendix Fig S4B for details on the molecular characteristics of these tumors).

APPENDIX SUPPLEMENTAL METHODS

Flow Cytometry

To measure the effects of the in vivo treatment with Nultin-3 and Paclitaxel, used as monotherapies or in combinatorial regimens, cells dissociated from Numb$^-$ and Numb$^+$ tumors were subjected to Aldefluor assays and co-stained with APC-CD44 and PE-CD24 antibodies, or stained with Dye eFluor®450 (eBioscence) to assess cell viability. Only lived cells were analyzed. The Aldefluor assay was performed using the manufacturer's protocol (ALDEFLUOR kit, StemCell Technologies, Durham, NC, USA). Briefly, one-million single cell suspensions were incubated in Aldefluor buffer containing the ALDH protein substrate (BAAA, BODIPY-aminoacetaldehyde, 1 mmol/L) for 45 minutes at 37°C. Cells that could catalyze BAAA to its fluorescent product (BAA) were considered ALDH+. Sorting gates for FACS were drawn relative to cell baseline fluorescence, which was determined by the addition of the ALDH-specific inhibitor diethylaminobenzaldehyde (DEAB) during the incubation. For CD44 and CD24 stainings, cells were resuspended in a 100 µL staining volume of FACS buffer (HBSS + 3% bovine serum albumin), incubated on ice with CD44-APC (BD Biosciences) or CD24-PE (BD Biosciences), according to the manufacturer's recommended protocol, and kept on ice for 40 min. The Dye eFluor®450 (eBioscence) or DAPI was used to measure viable, apoptotic and dead cells that were excluded from the analysis. Duplicates and dead cells were also excluded by gating with FSC and SSC. Cells were analyzed in a FACS Attune (Life Technologies) and the acquisition and analysis software were Attune Nxt 2.5 and Kaluza analysis 1.5A. Sorting gates for FACS were drawn relative to cell baseline fluorescence of isotype controls.

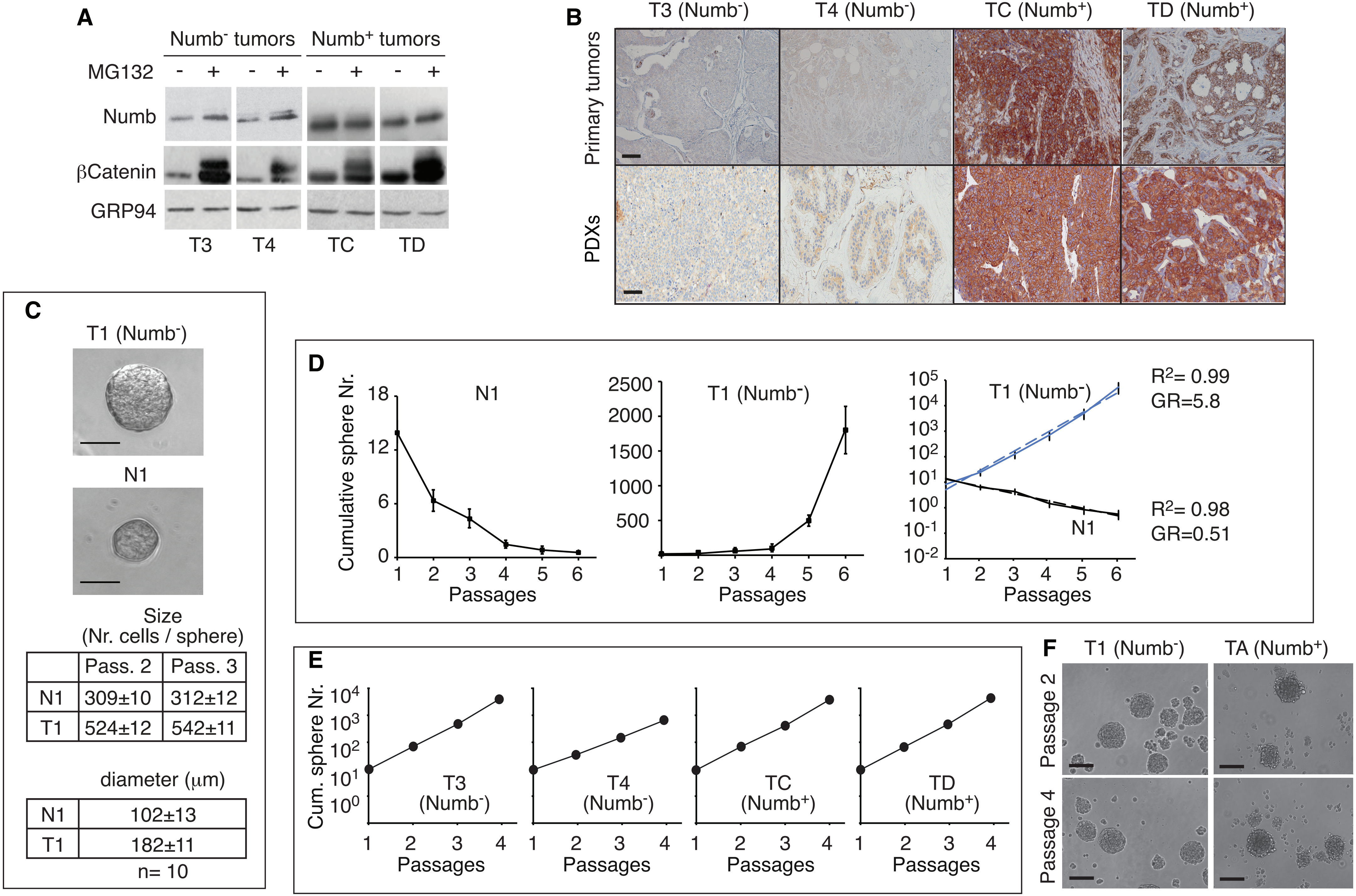

Appendix Figure S1

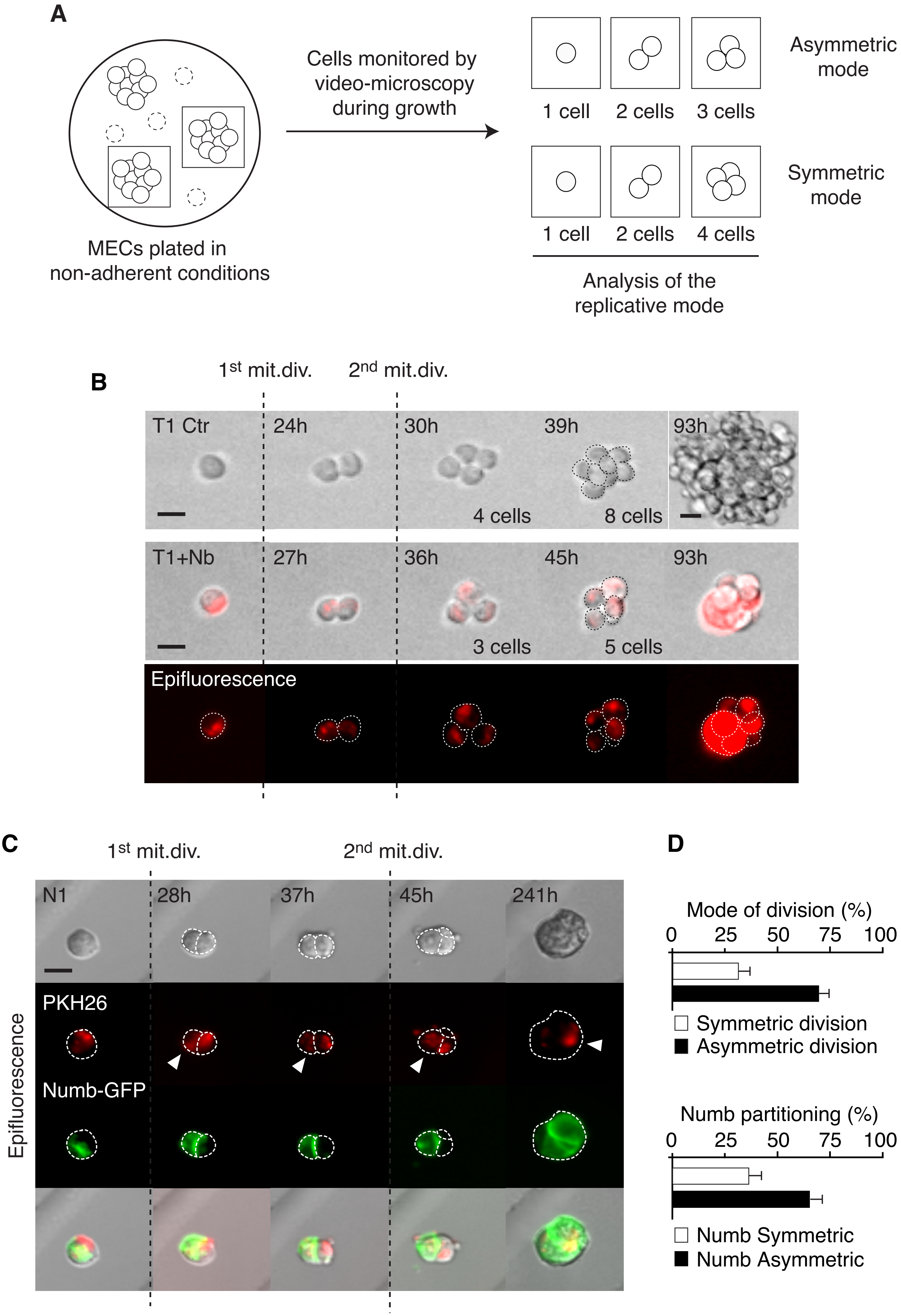

Appendix Figure S2

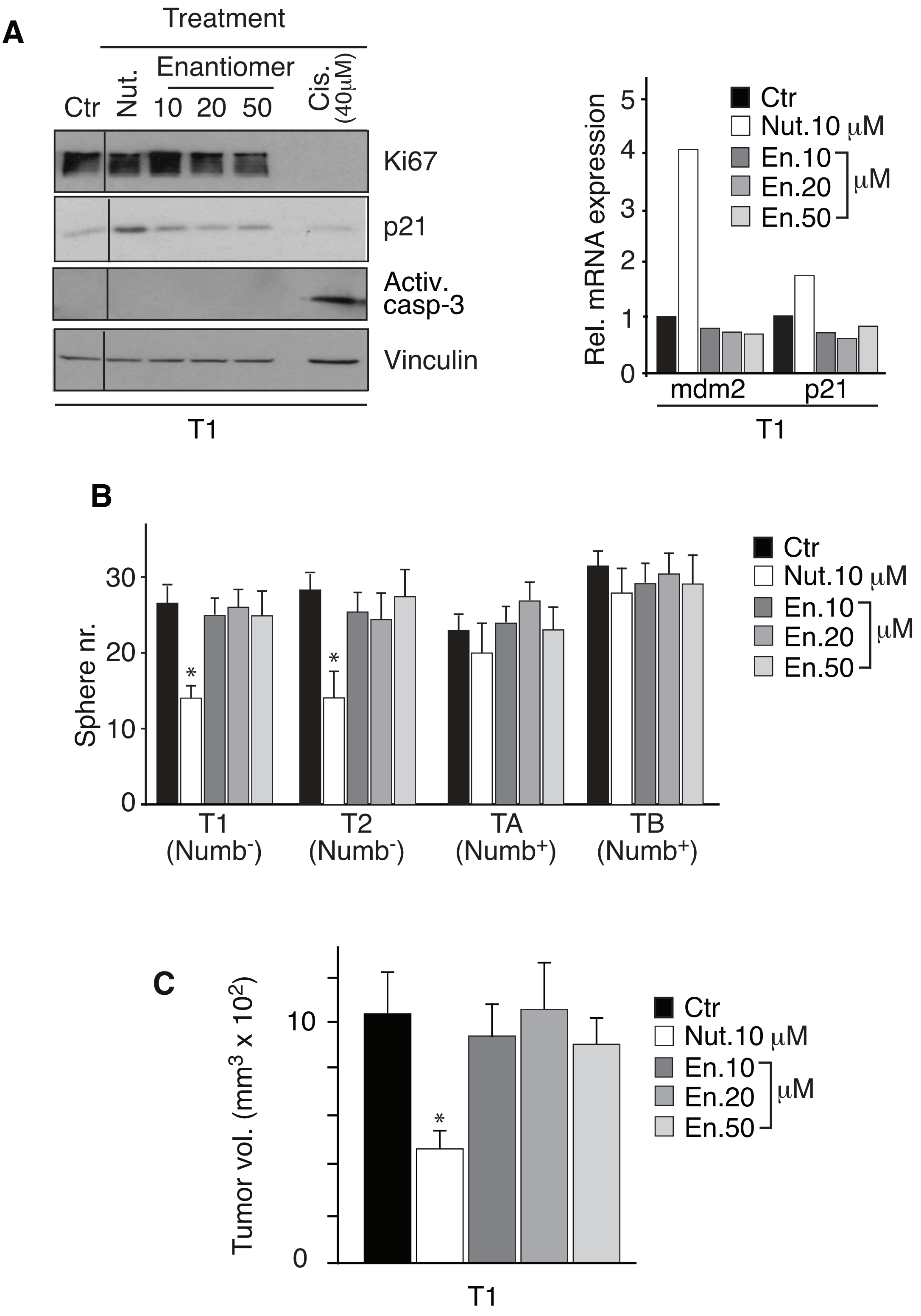

Appendix Figure S3

A

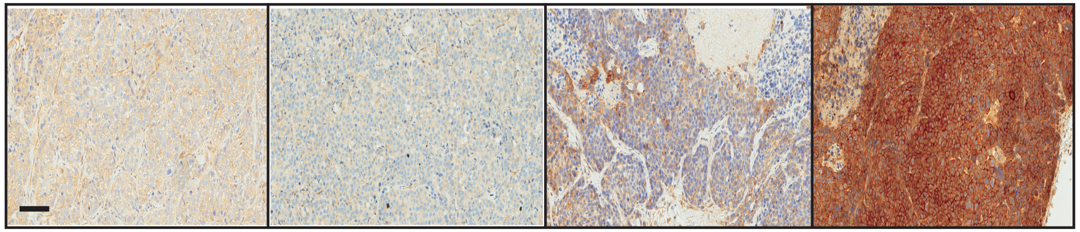

B

Tumor Name	Numb Status	Grade	HER2	ER%	PR%	p53 Status
T5	Numb$^-$	G3	NEG	0	0	WT
T6	Numb$^-$	G3	NEG	0	0	WT
T7	Numb$^-$	G3	NEG	90	90	WT
TE	Numb$^+$	G3	NEG	0	0	WT

C

			Ki67	Act.casp-3
Numb$^-$ tumors	T5	Vehicle	82%	<2%
	T5	Nut	78%	<2%
	T5	Chemo	35%	5.4%
	T5	Chemo+Nut	33%	7.4%
	T6	Ctr	67%	<1%
	T6	Nut	72%	<1%
	T6	Chemo	52%	<5%
	T6	Chemo+Nut	37%	<5%
	T7	Vehicle	88%	<2%
	T7	Nut	84%	<2%
	T7	Chemo	57%	5.2%
	T7	Chemo+Nut	51%	4.2%
Numb$^+$ tumor	TE	Vehicle	43%	<2%
	TE	Nut	37%	<2%
	TE	Chemo	25%	4.7%
	TE	Chemo+Nut	23%	3.8%

D

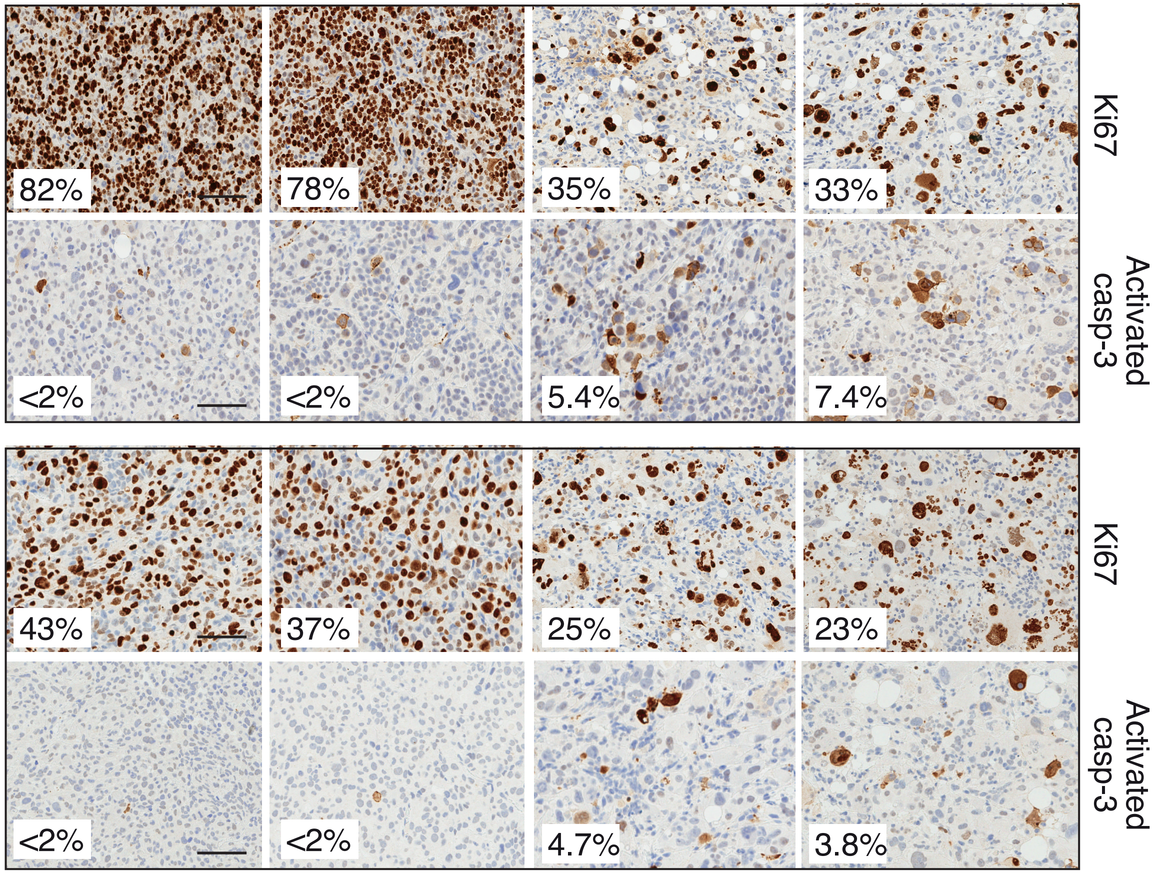

Appendix Figure S4

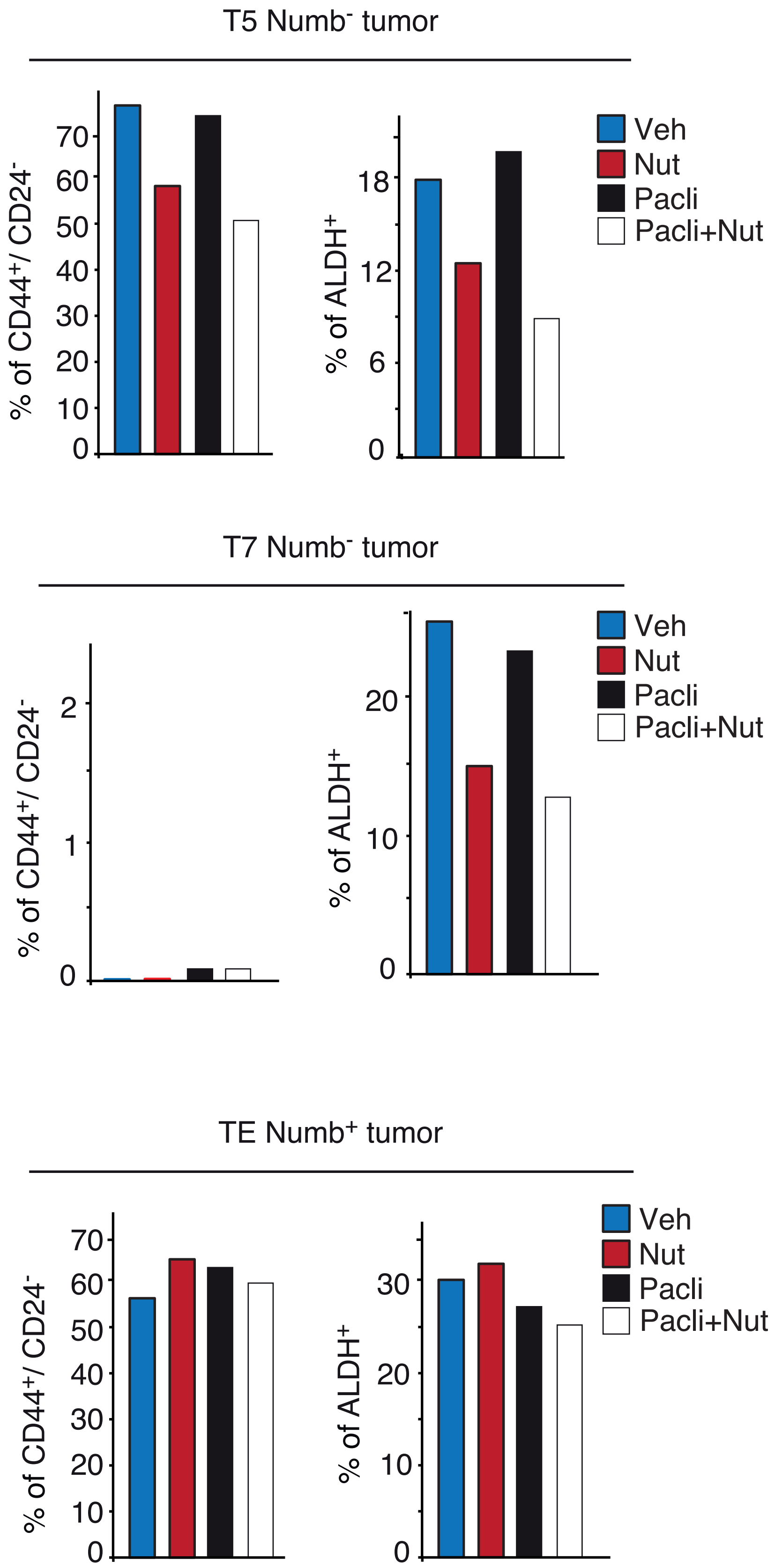

Appendix Figure S5
